# A comprehensive review of adherence to diabetes and cardiovascular medications in Iran; implications for practice and research

**DOI:** 10.1186/2251-6581-12-57

**Published:** 2013-12-20

**Authors:** Amir Sarayani, Zahra Jahangard-Rafsanjani, Molouk Hadjibabaie, Alireza Ahmadvand, Mohammadreza Javadi, Kheirollah Gholami

**Affiliations:** 1 Research Center for Rational Use of Drugs, Tehran University of Medical Sciences, Tehran, Iran; 2 Faculty of Pharmacy, Tehran University of Medical Sciences, Tehran, Iran; 3 School of Public Health, Tehran University of Medical Sciences, Tehran, Iran

**Keywords:** Medication adherence, Patient compliance, Cardiovascular diseases, Hypertension, Diabetes mellitus, Oral hypoglycemic medications, Patient education, Iran

## Abstract

Treatment of diseases such as diabetes mellitus and cardiovascular disorders are highly dependent on medications and particularly adherence to medications to achieve optimal pharmacotherapy outcomes. Several factors can affect a patient’s adherence including: knowledge and beliefs about their illness and medications, concomitant psychological disorders, type of therapeutic regimen, and lack of access to medicines. In Iran, a middle income country, essential medicines are highly available and affordable. However, adherence to medications has not been emphasized especially for patients with diabetes and cardiovascular diseases. In the present study, we reviewed the available literature on adherence to medications used to treat diabetes and cardiovascular disorders in Iran. We systematically searched Scopus, Web of Science, PubMed, CINAHL, Google Scholar, Scientific Information Database, and IranMedex using a highly sensitive protocol on July 2012. We retrieved 1003 citations; and two independent researchers screened them for relevant publications. Studies were included if they reported rate or determinants of adherence to diabetes mellitus and cardiovascular medications. Trials on improving interventions were also included. The quality of studies was assessed using appropriate guidelines. Fourteen studies were eligible for data extraction and review. The definition of adherence and the measurement tools used were unclear among studies. Methodological caveats including inappropriate sample size, sampling methods, inclusion/exclusion criteria, and high rate of loss to follow-up were also observed. Nevertheless, adherence rate was reported to be 62.8-86.3% for oral hypoglycemic medications and 38.8-60.0% for cardiovascular medicines. Forgetfulness, lack of knowledge about medical condition and prescribed medications, and concerns about medications efficacy and side effects were consistently reported as barriers to adherence. Patient education plus telephone or short message service follow-ups were reported to improve adherence to oral hypo-glycemic medications. We did not find any high quality trials on adherence to cardiovascular medicines. In conclusion, adherence to cardiovascular and diabetes medications is not assured in Iranian patients. Based on the available literature, patient education and reinforcement interventions are required to address this issue. Future studies should employ careful designs and standard tools for assessment of adherence to medications.

**Electronic supplementary material:**

The online version of this article (doi:10.1186/2251-6581-12-57) contains supplementary material, which is available to authorized users.

## Introduction

Medicines play an important role in medical care; and adherence to medications (AM) is essential to achieve best possible pharmacotherapy outcomes [1, 2]. Although there is no consensus on the ideal rate of AM in medical literature, anecdotal evidence suggests a rate of 80% to be adequate [3]. Some studies suggest that a limit of 95% should be considered as an acceptable AM rate particularly for certain medical conditions such as HIV/AIDS [4, 5]. Evidence shows that non-adherence to medications results in higher health care costs, longer hospitalizations, and increased morbidity and mortality [6–8].

Non-adherence to medications in patients with diabetes mellitus (DM) and cardiovascular diseases (CVD) is of major concern [9, 10]. In Iran, prevalence of type 2 DM is estimated to be 24% in adults aged over 40 years [11]. Furthermore, total direct cost of type 2 DM in 2009 was estimated to be 2.04 billion US dollars [12]. In addition, CVD is ranked as the third most important cause of disease and injury in the country [13]. Among CVD, hypertension prevalence is estimated to be 17% while significant rates of undiagnosed or uncontrolled cases have been reported [14]. Unfortunately, AM has not been highlighted for Iranian patients with DM or CVD and few studies have focused on the rate and the determinants of adherence [15, 16].

Adherence to medications is a complex behavior which can be influenced by patient, provider, and health system factors [17]. Numerous factors including cognitive impairments, adverse drug reactions, lack of knowledge about illness and pharmacotherapy regimen, complexity of the regimen and poor access to medicines have been identified to affect AM [18, 19]. Several interventions including reminder systems, follow-up programs by health care providers, and information technology tools have been developed to overcome patient and health care provider-related barriers [20]. Nevertheless, addressing the health system related factors such as access to medicines requires macro level policy making particularly in resource-limited settings [21]. In contrast to many other developing countries, access to medicines in Iran is reported to be addressed adequately through local production of generic medicines [22]. However, achieving optimal clinical outcomes requires patients’ adherence with the therapeutic regimen.

In the present study, we performed a comprehensive and systematic review of the available literature to identify the rate and the determinants of adherence to DM and CVD medications in Iran. We also reviewed publications on adherence to medications improving interventions in patients with DM and CVD.

## Methods

We searched international biomedical databases including Scopus, Web of Science, PubMed, CINAHL, and Google Scholar. National electronic databases including Scientific Information Database (SID) and IranMedex were also searched for Farsi or English language publications.

### Search strategy

We used MeSH terms “Medication Adherence” or “Patient Compliance” and other text words to develop our search protocol. Text words related to medicines included “medication, medicines, drug, therapy, treatment, and regimen” and keywords related to AM were “compliance, adherence, compliant, adherent, nonadherence, noncompliance, nonadherent, and noncompliant”. We used “Iran or Iranian” to restrict our search in international databases to publications related to Iran. National databases were searched using English keywords and their Farsi equivalent terms. To reach maximum sensitivity in our search protocol, we did not restrict for disease type. No restrictions were set for either time or type of publications. All database searches were carried out on July 2012.

### Study selection

All records retrieved from international databases were imported into a bibliographic software (EndNote® X5) library. Two independent researchers screened title and abstract of each record to find relevant publications and disagreements were resolved by discussion. Records in national databases were screened online as no export option was available for bibliographic software.

All articles which had reported the rate or score of adherence to DM or CVD medications in Iran were included in the study. Studies which had focused on AM determinants or AM improving interventions were also included. Studies were excluded if AM was reported as part of a clinical trial since patients were intended to have acceptable AM in such studies. After the screening phase, the full-text of relevant articles was retrieved if available online or through contact with corresponding authors. We contacted all the corresponding authors to ask for any unpublished data or any publications not retrieved during the bibliographic search.

### Data extraction and analysis

Two independent teams of researchers reviewed the full-text articles according to the inclusion–exclusion criteria. If articles were eligible for the study, reviewers extracted data on study characteristics and outcomes using a standardized extraction chart. Specific information on study design (randomized controlled trial, cohort, cross-sectional, qualitative), study setting, AM definition and rate, AM measurement tool (self-report, pill count, refill data, electronic medication monitors, or biological assessments), and AM determinants were recorded. Disagreements were resolved by discussion. Quality assessment was performed using an adapted version of STROBE checklist for cross sectional studies and CASP tools for clinical trials and qualitative studies (each of the checklists consisted of ten items-10 points) [23, 24]. Studies were categorized as low (0–3 points), moderate (3–7 points) and high (7–10 points) quality by two independent teams of researchers and disagreements were resolved by discussion. The low quality studies were excluded and data were extracted from moderate and high quality studies. We did not employ a meta-analysis approach as AM definitions and measurement tools were highly diverse among studies.

## Results

### Overview

**Figure 1:**
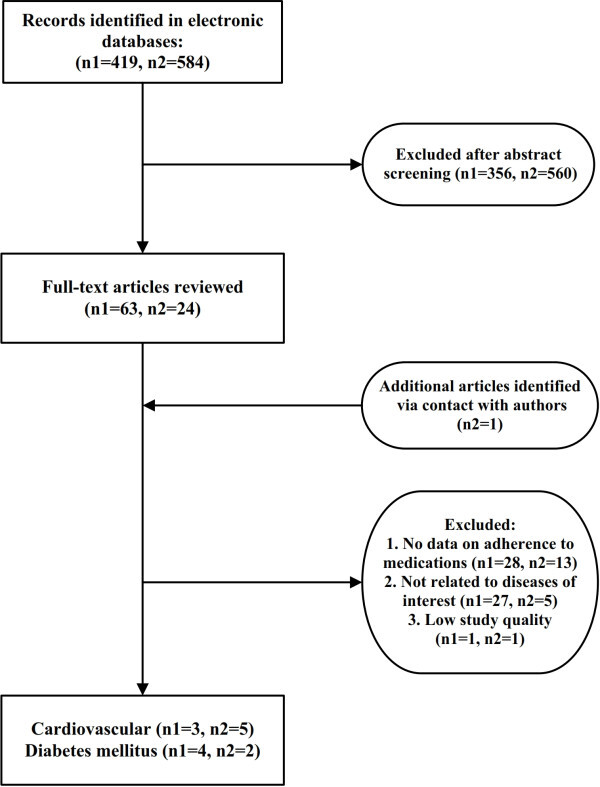
**Flow diagram of study selection.** n1: number of records in international database, n2: number of records in national database.

Our study retrieved 1003 citations of which 14 were eligible for data extraction and review (Figure 1). Of 14 publications, six articles were related to DM and eight articles were related to CVDs. Six out of 14 publications were in Farsi language and were retrieved from national databases. Adherence to medications was the primary objective in eight studies. A majority of publications were reports of cross-sectional studies (8 articles). A summary of studies is provided in Tables 1 and 2.

**Table 1:** **Summary of studies on adherence to diabetes medications**

Author/language(En/Fa)^a^	Publication year	Study design	Study population/ location	Sample size	Study aim/type of intervention	Adherence definition	Adherence measurement tool	Adherence rate/score	Study quality^c^
Aflakseir^31^ (En)	2012	Cross-sectional	Type 2 diabetes An outpatient clinic Shiraz	102	To examine the role of illness and medication perceptions on medication adherence in a group of Iranian patients with type 2 diabetes	“The score higher than midpoint has been considered as the index of adherence”	Self-report questionnaire (MARS^b^)	Adherence rate: 87%	***
Zolfaghari et al. ^27^ (En)	2011	Quasi-experimental (Randomized trial)	Type 2 diabetes Iranian Diabetes Association Tehran	77	Three-day live education plus:	Not Defined	Self-report questionnaire/11 items	Pre-test/Post-test scores (max. 100): SMS: 73.27/94.73 Telephone: 75.48/91.13	***
					1. Short message service (SMS) 2. Telephone follow-up by nurse				
Alizad et al. ^33^ (Fa)	2011	Qualitative	Insulin dependent diabetic patients (type 1 & 2)	64	To investigate the determinants of non-adherence to insulin therapy in type 1 and 2 diabetic patients	Not Defined	Not Defined	Not Applicable	***
			Medical University-affiliated DM centers of Tehran, Tabriz, Rasht, and Mashhad						
Farsai et al. ^15^ (En)	2011	Cross-sectional	Type 2 diabetes Research Center Clinic Isfahan	248	To determine the adherence to oral hypoglycemic medications and associated factors	“Patients who take 90-105% of medications are considered as adherent”	Self-report questionnaire/Pill count	Adherence rate: 62.3%/62.8%	**
Nesari et al. ^28^ (En)	2010	Randomized controlled trial	Type 2 diabetes Iranian Diabetes Association Tehran	61	Three-day live education plus:	Not Defined	Self-report questionnaire/7 items	Pre-test/Post-test scores(max. 100): Intervention: 61.11/89.55	***
					1-Telephone follow-up by nurse 2- Usual care				
								Control: 75.66/78.00	
Dabaghian et al. ^26^ (Fa)	2005	Cross-sectional	Type 2 diabetes Two tertiary hospitals Tehran	256	To determine the medication adherence rate and its association with patients knowledge and attitude about diabetes care	Compliance=[(N-n)/N]*100 N: Number of prescribed medications	Pill count	Adherence rate:	**
								Good: 86.3% Fair: 7% Bad: 6.6%	
						n: Number of consumed medications			
						C > 90% : Good			
						80 < C < 90 : Fair			
						C < 80 : Bad			

^a^En: English, Fa: Farsi, ^b^Medication adherence reporting scale, ^c^Quality rating: *Low, **Moderate, ***High.

**Table 2:** **Summary of studies on adherence to cardiovascular medications**

Author/language(En/Fa)^a^	Publication year	Study design	Study population/location	Sample size	Study aim/type of intervention	Adherence definition	Adherence measurement tool	Adherence rate/score	Study quality^e^
Heydari et al. ^29^ (En)	2011	Cross-sectional	Heart Failure	108	To investigate the relationship of a psychological model with adherence to therapeutic regimen	Not Defined	Self-report questionnaire/4 items	Adherence score 56.9 (max. 100)	***
			Two tertiary hospitals						
			Mashhad						
Heydari et al. ^35^ (Fa)	2011	Cross-sectional	CVD^b^	600	To determine the frequency of rehospitalization and its contributing factors	“Stop drug intake or irregular intake in second admission group”	Self-report questionnaire/6 items	Not Applicable	***
			Five hospitals						
			Mashhad						
Mohammadi et al. ^36^ (Fa)	2006	Randomized controlled trial	Hypertension One hospital Ardabil	200	Face to face education and a follow-up program using home visits for 3 months	“Regular intake of medications”	Not Defined	Adherence rates:	**
								Pre-test/Post-test:	
								Control group: 35.87%/42.4%	
								Intervention group 39.13%/44.6%	
Hadi et al. ^30^ (En)	2006	Randomized controlled trial	Hypertension Outpatient Clinic Shiraz	150	A four-arm trial, Live training plus one of the following reinforcement methods: (1) Telephone follow-up (2) Telephone follow-up plus educational booklet (3) educational booklet only (4)Usual care	Adherence score was calculated. (range: 0–5)	Self-report questionnaire/5 items (Modified MMAS^d^)	Adherence score :	**
								Pre-test/Post-test	
								(1) 2.67/4.14	
								(2) 2.25/3.88	
								(3) 2.82/4.35	
								(4) 2.92/4.23	
Abbasi et al. ^32^ (Fa)	2005	Cross-sectional	Hypertension Tehran Heart Center Clinic Tehran	380	Identification of compliance rate to drug regimens and its association with patients’ health beliefs	Adherence:	Self-report questionnaire (MMAS)	Adherence rate: 60%	**
						0-1 : Good 2-4 : Poor			
Hadi et al. ^16^ (En)	2004	Cross-sectional	Hypertension	250	To investigated factors associated with medication compliance among hypertensivepatients	“proportion of amount of drugs used by patients compared to the amount of drugs, which had been prescribed” >90% = good compliance	Self-report questionnaire	Adherence rate: 39.6%	***
			Outpatient Clinic						
			Shiraz						
Parsa-Yekta et al. ^25^ (Fa)	2004	Cross-sectional	CAD^c^ Outpatient clinic Tehran	150	To identify factors related to medication compliance	“percent of taken tablets” Good: 95-100% Moderate: 90-95% Weak: 56%	Pill count	Adherence rate:	***
								Pill count:	
								Good: 28%	
								Weak: 56%	
Mohammadi et al. ^34^ (En)	2002	Qualitative (Grounded Theory)	Hypertension	12	To identify the conceptual structure of high blood pressure control in an Iranian hypertensive population (to develop a theoretical explanation for the issue)	Not Defined	Not Applicable	Not Applicable	***
			Patients, Physicians, and Nurses were interviewed						
			Location unclear						

^a^En: English, Fa: Farsi, ^b^Cardiovascular diseases: heart failure, ischemic heart disease, cardiac arrhythmia, deep vein thrombosis, cardiac valve diseases. ^c^Coronary artery disease: myocardial infarction, angina pectoris. ^d^Morisky medication adherence scale, ^e^Quality rating: *Low, **Moderate, ***High.

### Adherence definition

The definition of adherence was highly diverse and unclear among studies. Four studies had operationalized adherence definition as “percent of medications consumption” [15, 16, 25, 26]. They had categorized adherence rate as good, fair, or poor; but the cut off thresholds were not exactly comparable. Four studies had used “adherence score” which was calculated on the basis of self-report questionnaires [27–30].

### Adherence measurement tools

Self-report approach was employed in 11 quantitative studies; however, different measurement tools were used. Three studies had utilized translated versions of standard AM assessment questionnaires including “Morisky Medication Adherence Scale (MMAS)” and “Medication Adherence Rating Scale (MARS)” [30–32]. In other studies, questionnaires were developed according to the study objectives. Few studies used pill count technique [15, 25, 26].

### Summary of studies

#### Diabetes mellitus

Three studies investigated the effects of demographic factors, knowledge of disease and belief about medications on adherence to oral hypoglycemic agents [15, 26, 31]. Self-reported causes for non-adherence to medications were reported in two of the studies (Table 3) [15, 26]. One study showed that adherent patients achieved an improved clinical outcome (HbA1c) in comparison with their non-adherent counterparts (7.1% ±1.2% vs. 7.8% ±1.3% for metformin users and 7.2% ±1.2% vs. 7.9% ±1.4% for glyburide users, respectively) [21]. Adherence to insulin therapy was investigated in one qualitative study [33]. Findings revealed five categories of AM determinants: (1) fear of insulin injection due to pain and blood (2) disturbance of daily life (3) negative attitudes about insulin side effects and its stigmatization (4) lack of proper training (5) impaired physical and financial competence.

**Table 3:** **Determinants of adherence and self-reported causes of non-adherence to diabetes medications**

Determinants of adherence	
Factor	Effect
Age	(−); r= −0.2 [31]
	(+); >45 years, p<0.001 [26]
	(×)[15]
Gender	(×) [15, 26]
Education level	(×) [26, 31]
	(+); p=0.007 [15]
Duration of pharmacotherapy	(×); [15, 26, 31]
Beliefs about medications	(−); r= −0.44 (concerns) [31]
	(+); p=0.009 (positive attitude) [26]
Knowledge of the disease	(+); p=0.01 [26]
**Self-reported causes for non-adherence**	
**Cause**	**Frequency**
Forgetfulness	38% [15]
	27% [26]
Medications not available	15.2% [26]
Regimen Complexity	15.1% [15]
Fasting during Ramadan	11% [15]
Feeling well/Lack of symptoms	7% [26]

(+) shows a direct/positive association or correlation (−) shows an inverse/negative association or correlation, (×) shows no significant relationship.

Few studies had evaluated interventions to improve AM in Iranian diabetic patients. Two studies evaluated nurse-led interventions using telephone or cell phone text messages following diabetes training workshops [27, 28]. Telephone follow-up consisted of 16 phone calls and the text message intervention utilized 72 messages during 3 months. Both studies revealed significant improvements of AM scores in the intervention groups comparing to usual care.

#### Cardiovascular diseases

Three studies had evaluated the determinants of adherence to medications in cardiovascular diseases (Table 4) [16, 25, 32]. Another study evaluated the relationship between a psychological model and adherence to hypertension therapeutic regimen (diet, exercise, and medications). Although, a distinct score was reported for AM, the determinants of adherence were analyzed for the therapeutic regimen as a whole [29]. A grounded theory-based study described the deficiencies of hypertension care (including medications) for Iranian patients [34]. The study suggested a “Partnership Care Model” in which non-adherence was in a reciprocal relationship with lack of knowledge and lack of effective care. One study reported that patients who were adherent to their medications had significantly lower systolic and diastolic blood pressures (mean difference: 10.2 and 5.1 mmHg, respectively) [16]. In another study, non-adherence or non-persistence to medications were identified as a main cause of rehospitalization (23% of cases) for CVD patients [35].

**Table 4:** **Determinants of adherence and self-reported causes of non-adherence to cardiovascular medications**

Determinants of adherence	
Factor	Effect
Age	(+); >50 years, p= 0.01 [16]
	(−); p<0.005 [25]
Education level	(+); p<0.005 [25]
Insurance coverage	(+); p= 0.01 [16]
	(×) [25]
Employment	(+); p< 0.005 [25]
Duration of pharmacotherapy	(+); <1 year, p= 0.002 [16]
	(−); p<0.005 [26]
Number of medications	(×); p= 0.78 [16]
Beliefs about medications	(+); p=0.006 [16]
	(×); [25]
Perception of disease severity	(+); p= 0.01 [32]
Perception of barriers to medications use	(+); p< 0.001 [32]
Knowledge	of disease (+); p= 0.01 [6]
	of medications (+); p< 0.005 [25]
Regular visits with physician	(+); p=0.001 [16]
**Self-reported causes for non-adherence**	
**Cause**	**Frequency**
Forgetfulness	30.1% [25]
Lack of symptoms	20.4% [25]
Side effects	15.6% [25]
Lack of efficacy	9.6% [25]

(+) shows a direct/positive association or correlation (−) shows an inverse/negative association or correlation, (×) shows no significant relationship.

We found no high quality trials on AM improving intervention for CVD patients. However, two studies reported the effect of training and follow-up interventions for hypertensive patients [30, 36]. Hadi et al. reported that face to face training plus educational booklet alone, weekly telephone follow-up, or weekly telephone follow-up and educational booklet are all effective strategies in improving AM. Nevertheless, no significant difference was observed between study groups (61% loss to follow-up) [30]. In contrast, Mohammadi et al. evaluated the impact of a program consisted of face to face training session at clinic and monthly home visits. The authors reported that the program was not significantly successful in comparison with the control group. However, ambiguity exists in the definition of AM and outcome assessment tool of the study [36].

## Discussion

In the present review, we encountered a range of studies regarding AM definition, measurement tools, and method of reporting (rate vs. score). Nevertheless, some studies have reported adherence rates of 62.3% to 87% among diabetic patients and 28% to 60.0% among patients with CVD in Iran [15, 25, 31, 32]. These findings are comparable with international literature reports of AM rates to be 50-70% for various medical conditions [2, 9, 21]. Despite methodological issues which will be discussed later in this section, insufficient rate of AM is evident for both DM and CVD medications in Iran. Moreover, we found two studies which reported the association of clinical outcomes (lower HbA1c and blood pressure levels) with higher adherence rate to DM and CVD medications in Iranian patients [15, 32]. Such findings are also in line with other studies which have revealed the association of AM with improved clinical outcomes and lower morbidity and mortality rates [37–40].

Patient-related factors including demographic characteristics, knowledge and beliefs about illness and medications, concomitant psychological disorders, and therapeutic regimen characteristics have been mostly linked to nonadherence behavior [18, 40, 41]. In Iranian patients, factors such as age, education level, duration of pharmacotherapy, and insurance coverage did not influence AM consistently ( i.e. positive, negative or neutral associations have been reported) [15, 16, 25, 26, 31, 32]. In contrast, patients’ knowledge of their illness or medications and their beliefs about medications efficacy or side effects were reported to adversely affect AM in all studies [16, 25, 26, 31, 32]. Forgetfulness, lack of symptoms, fasting during Ramadan, lack of efficacy, and fear of side effects were among self-reported causes of non-adherence to medications [15, 25, 26]. Quantitative studies as well as qualitative studies have documented the importance of appropriate knowledge of and positive attitudes toward medications effect [33, 34]. Based on our review, it can be concluded that the lack of appropriate knowledge and negative attitudes is the most important barrier to adherence for patients with DM and CVD. Patient education programs are required to ensure optimal adherence rates. Patients reported forgetfulness as the most frequent cause of non-adherence. Forgetfulness could be addressed by modification of dosing schedules based on patients’ daily routines and also the use of reminder systems such as programmed devices, special reminder pill packaging, and appointment/prescription refill reminders [20, 42–44].

We found few studies on interventions to improve AM in Iranian patients. In two studies, patient education plus telephone or short message follow-up services were evaluated for diabetic patients. Education alone was not able to improve AM but education plus any of the follow-up tools could significantly improve adherence and clinical outcomes [27, 28]. This is in line with the results of a recent systematic review concluding that patient education alone does not seem to be successful in improving AM in hypertensive patients [45]. Thus, patient education must be accompanied by continuous support and reinforcement to maximize the effect of interventions. Tele-communication technologies including different telephone and cell phone services can be utilized as valuable support tools.

### Implications for research

The definition of AM and the terminology used for various aspects of non-adherence are highly diverse in the literature. This weakness is also observed among Iranian publications. However, international initiatives have focused on standardizing concepts of AM research [1, 46, 47] and future studies are encouraged to comply with these research guidelines. There are different methods of measuring AM including pill count, pharmacological and biochemical markers, medical and dispensing records, self-report, and Medication Event Monitoring System (MEMS) [2]. In our review, almost all studies had used self-report or pill count method to measure AM. In Iran, DM and CVD medications can be obtained from pharmacies without restrictions. Such access to medicines may deteriorate the validity of pill count method. In addition, unstandardized self-report questionnaires neither guarantee the accuracy of AM measurement nor can assure the generalizability of the results among studies. However, lack of prescription refill databases and high cost of MEMS devices limit their application for AM research and clinical practice. We believe that self-report method may be considered as the most appropriate tool for measuring AM in clinical practice and research in Iran. Standardized self-report questionnaires such as Morisky Medication Adherence Scale and Hill-Bone Compliance Scale shall be adapted and validated for Iranian patients [48]. We also observed various methodological weaknesses including lack of sample size calculation, non-probability sampling method, small sample size, and undefined inclusion criteria in Iranian studies. Such limitations should be taken care of in future studies in order to guarantee the internal and external validity of the results.

## Conclusion

Although medicines are highly accessible in Iran, patient-related factors in particular, lack of knowledge and positive attitudes about the illness and medications have resulted in impaired adherence with DM and CVD medications. We strongly suggest that health care professionals consider patients’ non-adherence to medications as a principal underlying factor for non-improvement in clinical outcomes. Furthermore, health policy makers should consider impaired AM as a major issue which requires multidisciplinary policies and interventions involving clinicians and other health professionals including pharmacists and nurses to be addressed the issue. Based on the available evidence, interventions should focus on patient education and reinforcement.

## Authors’ original submitted files for images

Below are the links to the authors’ original submitted files for images. Authors’ original file for figure 1
