# Supplementary figures and images for: A comprehensive review of adherence to diabetes and cardiovascular medications in Iran; implications for practice and research
Source: J Diabetes Metab Disord. 2013 Dec 20;12:57. doi: 10.1186/2251-6581-12-57 (PMC7962547; doi:10.1186/2251-6581-12-57)

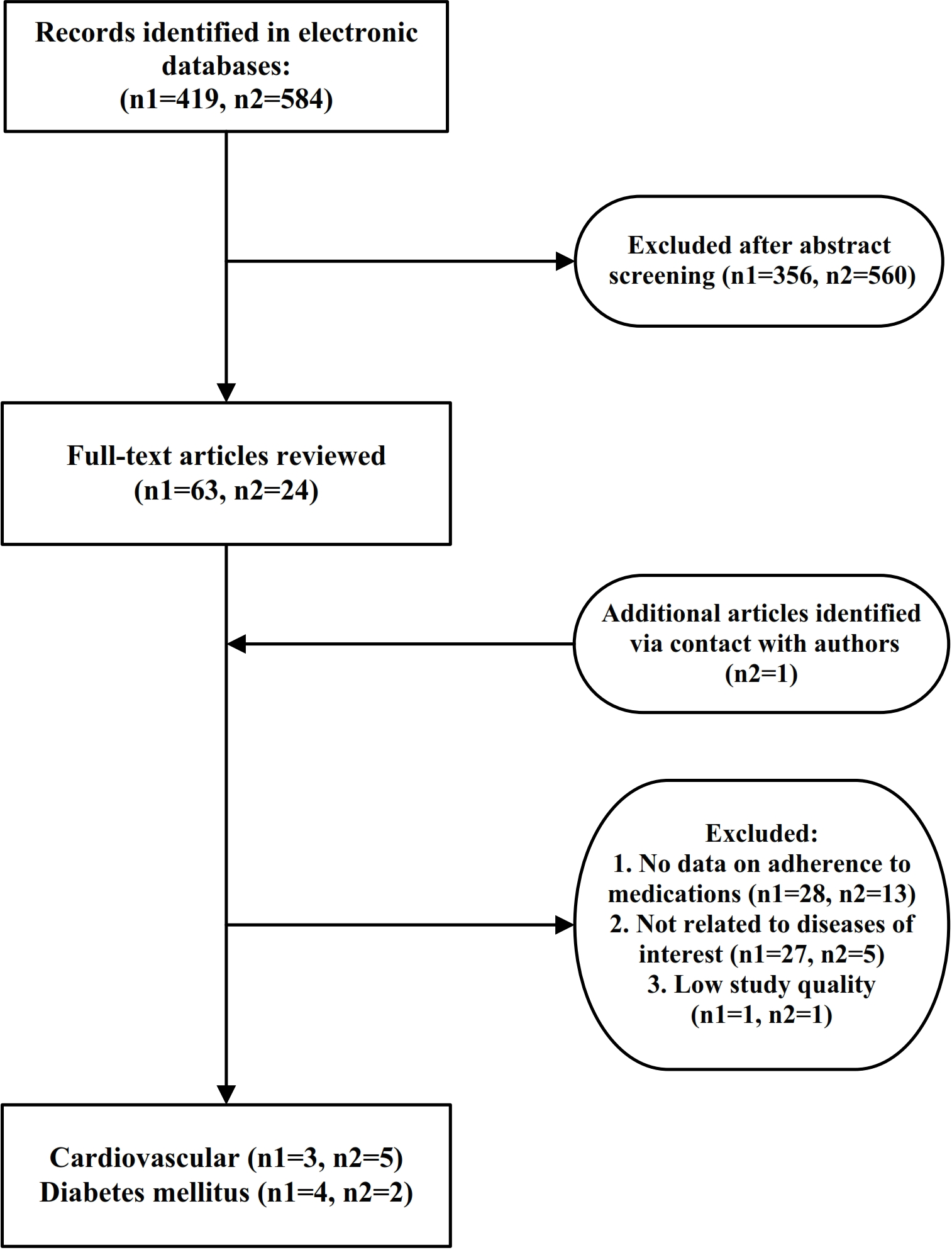

Supplement: Supplementary file 1 — Authors’ original file for figure 1 [file 40200_2013_74_MOESM1_ESM.tif]
